# In the absence of a “landscape of fear”: How lions, hyenas, and cheetahs coexist

**DOI:** 10.1002/ece3.2569

**Published:** 2016-11-06

**Authors:** Alexandra Swanson, Todd Arnold, Margaret Kosmala, James Forester, Craig Packer

**Affiliations:** ^1^ Department of EcologyEvolution and BehaviorUniversity of MinnesotaSaint PaulMNUSA; ^2^ Department of PhysicsUniversity of OxfordOxfordUK; ^3^ Department of Organismic and Evolutionary BiologyHarvard UniversityCambridgeMAUSA

**Keywords:** *Acinonyx jubatus*, carnivore coexistence, *Crocuta crocuta*, interference competition, *Panthera leo*

## Abstract

Aggression by top predators can create a “landscape of fear” in which subordinate predators restrict their activity to low‐risk areas or times of day. At large spatial or temporal scales, this can result in the costly loss of access to resources. However, fine‐scale reactive avoidance may minimize the risk of aggressive encounters for subordinate predators while maintaining access to resources, thereby providing a mechanism for coexistence. We investigated fine‐scale spatiotemporal avoidance in a guild of African predators characterized by intense interference competition. Vulnerable to food stealing and direct killing, cheetahs are expected to avoid both larger predators; hyenas are expected to avoid lions. We deployed a grid of 225 camera traps across 1,125 km^2^ in Serengeti National Park, Tanzania, to evaluate concurrent patterns of habitat use by lions, hyenas, cheetahs, and their primary prey. We used hurdle models to evaluate whether smaller species avoided areas preferred by larger species, and we used time‐to‐event models to evaluate fine‐scale temporal avoidance in the hours immediately surrounding top predator activity. We found no evidence of long‐term displacement of subordinate species, even at fine spatial scales. Instead, hyenas and cheetahs were positively associated with lions except in areas with exceptionally high lion use. Hyenas and lions appeared to actively track each, while cheetahs appear to maintain long‐term access to sites with high lion use by actively avoiding those areas just in the hours immediately following lion activity. Our results suggest that cheetahs are able to use patches of preferred habitat by avoiding lions on a moment‐to‐moment basis. Such fine‐scale temporal avoidance is likely to be less costly than long‐term avoidance of preferred areas: This may help explain why cheetahs are able to coexist with lions despite high rates of lion‐inflicted mortality, and highlights reactive avoidance as a general mechanism for predator coexistence.

## 1 Introduction

In ecosystems around the world, top predators kill, harass, and steal food from smaller predators (Caro & Stoner, 2003; Donadio & Buskirk, 2006; Linnell & Strand, 2000; Palomares & Caro, 1999). These direct, aggressive interactions, generally referred to as interference competition, are widespread and substantial, and can have profound consequences for the distributions and population dynamics of smaller predators (Prugh et al., 2009; Ritchie & Johnson, 2009). Top predators often suppress or exclude subordinate predators from the larger landscape (Kamler, Ballard, Gilliland, Lemons, & Mote, 2003; Newsome & Ripple, 2014; Swanson et al., 2014), and the loss of top predators can result in subordinate predator “release” or rapid population expansion (Prugh et al., 2009; Ritchie & Johnson, 2009); in turn, these dynamics can have widespread, cascading effects throughout the larger ecosystem (Estes et al., 2011; Ripple et al., 2014; Suraci, Clinchy, Dill, Roberts, & Zanette, 2016). However, these patterns of suppression and coexistence vary across systems and species (Elmhagen, Ludwig, Rushton, Helle, & Lindén, 2010; Elmhagen & Rushton, 2007; Swanson et al., 2014); thus, as top predators decline globally (Estes et al., 2011; Ripple et al., 2014), understanding the drivers of predator suppression and coexistence becomes increasingly important.

Spatiotemporal avoidance plays a critical role in minimizing the costs of interference competition and promoting predator–predator coexistence. Such avoidance takes many forms: Subordinate predators may avoid risky habitat types (Fedriani, Palomares, & Delibes, 1999; Mukherjee, Zelcer, & Kotler, 2008), hours of the day (Bischof, Ali, Kabir, Hameed, & Nawaz, 2014; Carothers & Jaksić, 1984; Hayward & Slotow, 2009), or known areas of apex predator activity (Kamler et al., 2003; Swanson et al., 2014). For example, foxes avoid habitats used by European lynx (Fedriani et al., 1999), and coyotes concentrate primarily at boundaries between wolf‐pack territories (Fuller & Keith, 1981). These avoidance strategies can be costly: By restricting their activity to “safe” areas or times of day in what is referred to as a “landscape of fear” (sensu Laundré, Hernández, & Altendorf, 2001), subordinate species can lose access to vital resources such as prey, water, or shelter (Lesmeister, Nielsen, Schauber, & Hellgren, 2015; Mukherjee et al., 2008; Ritchie & Johnson, 2009; Sergio & Hiraldo, 2008).

The demographic consequences of behavioral avoidance are well documented in small‐scale predator–prey systems (Preisser, Bolnick, & Benard, 2005; Schmitz, Beckerman, & O'Brien, 1997; Werner & Peacor, 2003) and can reasonably be expected to apply to predator–predator systems with interference competition. Indeed, large‐scale avoidance of top predator territories appears to be a primary driver of suppression of African wild dogs (Swanson et al., 2014) and swift foxes (Kamler et al., 2003). However, the strength of these costs depends on numerous factors such as predator hunting strategy (Schmitz, Krivan, & Ovadia, 2004) and habitat productivity (Bolnick & Preisser, 2005); considerable debate remains as to the relative importance of the landscape of fear in driving dynamics of large, wide‐ranging species (Beschta & Ripple, 2013; Kauffman, Brodie, & Jules, 2010).

Recent studies of large African predators have suggested that fine‐scale reactive avoidance strategies may promote coexistence by minimizing the risks of aggressive encounters but allowing subordinate species to maintain access to resources (Broekhuis, Cozzi, Valeix, McNutt, & Macdonald, 2013; Swanson et al., 2014). Instead of preemptively avoiding large portions of the landscape or preferred habitat types and losing access to the resources within, subordinate predator species may reactively alter their habitat use on a moment‐to‐moment basis. Fine‐scale avoidance may explain contrasting patterns of predator suppression documented in African carnivores (Swanson et al., 2014), highlighting a need to better understand how large predators share the landscape at fine spatial and temporal scales.

Here, we evaluate patterns of spatial and temporal avoidance among lions (*Panthera leo*), spotted hyenas (*Crocuta crocuta*), and cheetahs (*Acinonyx jubatus*) in Serengeti National Park, Tanzania. Despite dissimilar hunting strategies (Kruuk, 1972; Schaller, 1972), lions and hyenas show considerable dietary overlap (Hayward, 2006) and reciprocally harass and steal food from each other (Höner, Wachter, East, & Hofer, 2002; Kissui & Packer, 2004; Périquet, Fritz, & Revilla, 2015). Lions tend to dominate aggressive interactions and can suppress hyena populations through extensive food stealing (Watts & Holekamp, 2008). In large groups, hyenas can reciprocally displace lions from a carcass (Cooper, 1991; Höner et al., 2002) and directly benefit from these interactions (Watts & Holekamp, 2008). However, this does not appear to inflict a measurable cost to lions (Kissui & Packer, 2004), and lion and hyena population densities are positively correlated across African reserves (Creel & Creel, 1996). In contrast, lions and hyenas are both believed to suppress cheetah populations through high rates of direct killing (Laurenson, 1995) and cheetahs are often described as fugitive species, ranging widely and persisting only in marginal areas with low lion and hyena densities (e.g., Caro & Stoner, 2003; Chauvenet, Durant, Hilborn, & Pettorelli, 2011; Durant, 1998, 2000; Saleni, 2007). However, recent studies show that lion‐inflicted mortality is lower than previously assumed (Mills & Mills, 2014) and that cheetah populations are not suppressed by high lion densities (Swanson et al., 2014). Thus, while cheetahs actively avoid lions (Durant, 2000), considerable debate remains as to whether this response translates into long‐term spatial displacement that could threaten cheetah populations and conservation efforts (cf. Broekhuis et al., 2013 and Swanson et al., 2014 with Durant, 1998 and Vanak et al., 2013).

We use camera traps to evaluate fine‐scale spatial avoidance, characterized by long‐term (preemptive) avoidance of camera sites, and fine‐scale temporal avoidance, characterized by short‐term (reactive) avoidance of camera sites in the hours immediately surrounding top predator activity. Fine‐scale avoidance behavior has primarily been evaluated via extensive use of GPS collars (e.g., Broekhuis et al., 2013; Courbin et al., 2015; Vanak et al., 2013), but this can be prohibitively expensive. Camera traps provide an affordable alternative to simultaneously assess fine‐scale avoidance across multiple species, and we apply new methodological approaches to analyze camera‐trap data at finer temporal scales than allowed by standard multispecies occupancy models (MacKenzie et al., 2006; Richmond, Hines, & Beissinger, 2010; Sollmann et al., 2012).

We frame our analyses according to the respective competitive abilities of each species. As the dominant competitor, lions are expected to gain access to their preferred landscape characteristics, regardless of hyena or cheetah activity. Hyenas should be sensitive to lions, either avoiding lions because lions dominate aggressive interactions or following lions for scavenging opportunities. Because the outcomes of lion–hyena interactions depend on group size, this relationship may be complex. We expect cheetahs to avoid both lions and hyenas according to their “fugitive species” status.

## 2 Methods

### 2.1 Study system and field survey

**Figure 1:**
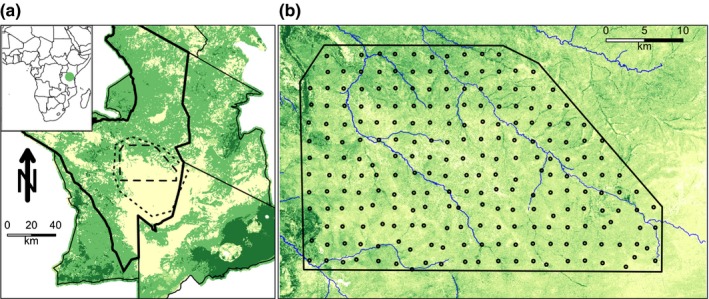
Study area. (a) Serengeti National Park (thick line) and surrounding protected areas (thin lines). Long‐term lion project study area in center is indicated by dotted line; camera‐trap study area is indicated by dashed line. Color reflects categorical ecosystem‐wide habitat designations of plains (yellow), open woodlands (light green), and closed woodlands (dark green). (b) Camera‐trap layout within the long‐term Lion Project Study Area. Camera locations are plotted over tree cover as extracted from Landsat imagery, with darker green indicating increased tree cover per 30‐m^2^ grid cell

Our 1,125‐km^2^ study area (bounded in the northwest at lat/long of −2.363589, 34.72594; in the southeast at −2.660651, 35.18051) was located at the center of Serengeti National Park, Tanzania, at the intersection of open plains and savanna woodlands (Figure 1a). Rainfall and vegetation follow a northwest–southeast gradient: wetter, denser woodlands in the northwest to drier, short‐grass plains in the southeast (Sinclair, 1995). The ecosystem holds some of the highest concentrations of large predators in the world (Sinclair, 1995) and is dominated by the annual migration of 1.6 million wildebeest (*Connochaetes taurinus*), zebra (*Equus quagga*), and gazelle (*Eudorcas thomsonii*) that follow the seasonal rains from Kenya's Masai Mara Reserve onto the nutrient‐rich plains of the Ngorongoro Conservation Area and Serengeti National Park (Holdo, Holt, & Fryxell, 2009).

The Serengeti Lion Project has monitored lion ranging and demography since the 1960s (Packer et al., 2005; Swanson et al., 2014). The camera survey is located within the long‐term lion study area, where all prides are monitored by radiotelemetry of one VHF‐collared female per pride.

We established 225 camera traps across a grid layout such that each camera was near the center of a 5‐km^2^ grid cell (Figure 1b). Cameras were set ~50 cm above ground level and covered a detection zone of ~15 m distance and 50° angle (Cusack et al., 2015). Operating continuously (aside from camera failure), the survey recorded 99,241 camera‐trap days between June 2010 and May 2013. Species, number of individuals, and behaviors in each image were identified by citizen scientists via *Snapshot Serengeti* (www.snapshotserengeti.org). Swanson et al. (2015) provide details on the camera survey and data processing. Although validation of volunteer classifications against an expert‐classified dataset demonstrates that final volunteer species classifications agree with experts 97% of the time (Swanson et al., 2015), we limited all analyses in this study to images with at least 75% agreement among raw classifications, guaranteeing 99.7% species identification accuracy (see Swanson, Kosmala, Lintott, and Packer (2016) for additional details).

### 2.2 Analytical approaches

#### 2.2.1 Spatial avoidance

We evaluated patterns of fine‐scale spatial avoidance among lions, hyenas, and cheetahs by comparing species‐specific capture rates at each site. Although raw camera‐trap capture rates do not reflect larger‐scale lion densities, likely due to lions’ attraction to isolated trees, comparison of camera‐trap captures to VHF‐collar lion locations indicates that cameras accurately reflect localized lion presence (Swanson, 2014). To ensure independence across captures, we limited analyses to no more than one sighting per day for a given species at a given site.

Because the camera‐trap data were zero‐inflated and overdispersed, we used hurdle models [also known as “zero altered Poisson/negative binomial” or ZAP/ZINB models (Zuur, Ieno, Walker, Saveliev, & Smith, 2009)] to specify two different underlying processes: a binomial process (species was detected or not) and a truncated count process [how many were seen (1 or more), *given that the species was detected*]. Because camera traps sample in only one direction and additional animals could have passed behind the camera, absence can imply true absence or lack of detection, and abundance represents minimum abundance.

For each subordinate species (hyenas and cheetahs), we evaluated a full model that included localized habitat characteristics, localized prey availability, and the localized presence/abundance of lions and the other subordinate species as predictors. For completeness and ability to compare joint habitat selection, we evaluated the same model for lions. We evaluated the relative importance of intraguild predators using analysis of deviance (ANODEV; Harris et al., 2005) and comparing models with and without covariates indicating the presence and abundance of intraguild predators. ANODEV was calculated as the difference in model deviance (−2logL) between the null model (no predictors) and the current model, divided by the difference between the null model and a full model that included all potential predictor variables. ANODEV reflects the fraction of model variation captured by the predictors in the current model, relative to the null model (which has a value of 0) and the full model (which has a value of 1).

The localized influence of each predator and prey species was included as *presence* in the binomial models (i.e., 1 if ever detected at that site; 0 if never detected at that site) and *abundance* in count models (i.e., number of independent captures at that site). The prey species included buffalo (*Syncerus caffer*), wildebeest (*Connochaetes taurinus*), and Thomson's gazelle (*Eudorcas thomsonii*) as the three main prey species of Serengeti lions, hyenas, and cheetahs, respectively (Schaller, 1972; Sinclair & Norton‐Griffiths, 1979). Habitat characteristics were as follows: Distance to river (m), Distance to kopje (m), Grass height (visually estimated on a scale of 1–5), Percent Tree Cover (<1 km, characterized from Landsat imagery by Sexton et al., 2013), Tree isolation (measured by the average distance (m) to the 10 nearest trees), Habitat (plains vs. woodlands), Shade (visually estimated on a scale of 0–4), and an interaction term for Tree isolation * Shade to reflect the potential attractiveness of an isolated shade tree. All nonbinary predictors were standardized by *z*‐score to facilitate comparing effect sizes across predictors with widely varying ranges and units (mean and standard deviations provided in Table S1). There was no significant colinearity among the predictor variables (*r* < 0.7 for all pairwise comparisons).

We evaluated patterns of partitioning aggregated across all years and controlled for site‐specific search effort by specifying an offset of log(# Camera‐trap days). To verify that patterns were robust within and among years, we aggregated all diurnal versus nocturnal sightings and all seasonal data (wet vs. dry season), as well as separately for each individual wet and dry season. In all cases, results were qualitatively similar to the overall aggregated analysis and are not reported here.

#### 2.2.2 Temporal avoidance

We summarized hourly detections of predator and prey species and interpreted statistically significant (*p *< .05) departures from an expected hourly frequency of 0.042 (1/24) as evidence of nonrandom diel activity patterns.

We evaluated temporal avoidance or attraction by calculating time since last prey (gazelle, wildebeest, or buffalo) and time since last predator (lion, hyena, or cheetah) for all independent predator sightings. Because many species will spend multiple hours at a site, we calculated all “time since” measures as the time difference between when the first species left and the second species arrived. We aggregated “time since” observations into thirteen 12‐hr bins (e.g., 0–12, 12–24, …, 132–144, or >144 hr since the last species) and assessed short‐term temporal attraction or avoidance by comparing observed versus expected number of observations during the first 0–12 and 12–24 hr following detection of another species. We calculated expected values for number of observations during each 12‐hr bin using a discrete time‐to‐event model that assumed exponential decay in the probability that the species of interest had not been seen yet, with expected encounter probabilities calculated using pooled information on detection probability throughout the first 6 days, with an adjustment based on probability of activity during each 12‐hr bin based on diel activity patterns for the second observed species. This assured that observed patterns were not due to similar or contrasting diel patterns (e.g., a strictly nocturnal species not observed in the 12 hr following an early morning observation of a mostly diurnal species might reflect temporal niche partitioning rather than active avoidance; see Swanson, 2014).

## 3 Results

### 3.1 Habitat use and spatial avoidance

**Figure 2:**
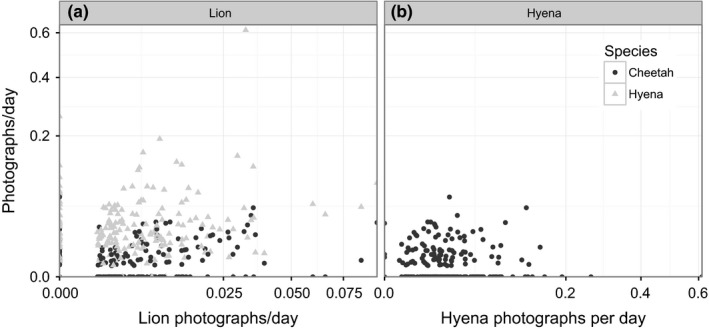
Photographic capture rates for subordinate versus dominant predators. (a) Cheetah and hyena capture rates (i.e., number of independent photographs per X days) plotted against lion capture rates for each site. (b) Cheetah capture rates plotted against hyena capture rates. Note that all axes are plotted on a square‐root scale

**Figure 3:**
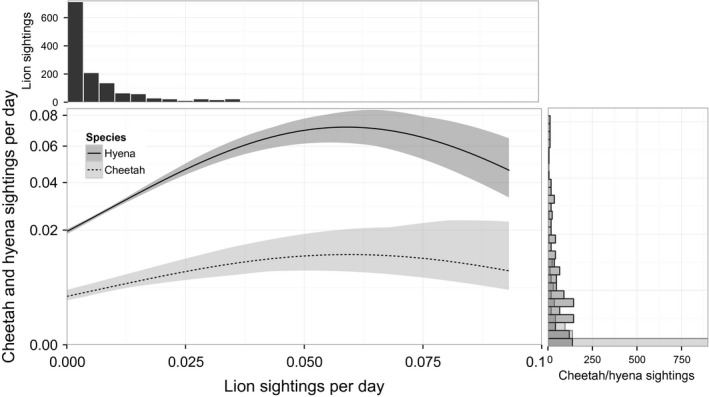
Predictions from full spatial models. Expected cheetah and hyena photographic capture rates as specified by full regression models, plotted against lion capture rates, holding all other parameters at their mean values. Histograms provide distributions of actual capture rates for lions (top) and hyenas and cheetahs (right)

Cheetah presence was highest at sites with shaded, isolated trees in relatively open habitat; of those sites where cheetahs were present, cheetah sightings were highest at shaded sites away from river confluences but closer to kopjes (Table 1a). Cheetah sightings also increased with lion sightings, reaching a maximum at approximately 0.06 lion sightings per day; although cheetah sightings declined slightly at sites with the highest lion numbers, confidence intervals overlapped broadly over a range of 0.025–0.1 lions per day (Figure 3). Although a significant predictor, lion abundance explained relatively little additional variation (8.6%) in cheetah presence and abundance after considering habitat and prey, which explained 88.0% of the total deviance (Table 2). Cheetah presence was not significantly related to hyena presence, but cheetah abundance exhibited a nonlinear relationship with hyena abundance (*p* = .006), declining when hyena abundance exceeded ~1.1 *SD* above average) (Table 1a; Figures 2 and 3). However, this effect was weak, and including hyenas in the spatial model explained only 3.4% more deviance (Table 2).

**Table 1:** Full models for presence and abundance of cheetahs, hyenas, and lions. All nonbinary coefficients were standardized via *z*‐score (mean and standard deviations provided in Table S1)

	Estimate	*SE*	*p*
(A) Full model: Cheetahs ~ Habitat, Prey, Lions, Hyenas			
Presence model: binomial with logit link			
(Intercept)	−3.991	1.336	0.0028**
Shade	0.881	0.249	<0.0001***
Tree isolation	1.118	0.303	<0.0001***
Percent Cover	−0.56	0.251	0.0256*
Distance to river	−0.028	0.258	0.9131
Distance to confluence	−0.247	0.278	0.375
Distance to kopje	0.077	0.189	0.6852
Habitat	−0.827	0.223	<0.0001***
Buffalo	0.327	0.529	0.5361
Wildebeest	2.256	1.203	0.0607
T. gazelle	0.181	0.829	0.827
Lion	0.593	0.421	0.1585
Hyena	1.087	0.929	0.2423
Shade*Tree isolation	0.688	0.276	0.0127*
Count model: truncated Poisson with log link			
(Intercept)	−5.342	0.145	<0.0001***
Shade	0.401	0.114	0.0004***
Tree isolation	0.15	0.096	0.1174
Percent Cover	−0.083	0.112	0.4556
Distance to river	−0.047	0.073	0.522
Distance to confluence	0.295	0.075	<0.0001***
Distance to kopje	−0.394	0.076	<0.0001***
Habitat	−0.225	0.15	0.1345
Buffalo/day	−0.13	0.104	0.2097
Wildebeest/day	−0.179	0.096	0.0636
T. gazelle/day	−0.162	0.116	0.1612
Lions/day	0.458	0.093	<0.0001***
Lions/day^2^	−0.055	0.014	<0.0001***
Hyenas/day	0.363	0.198	0.0661
Hyenas/day^2^	−0.331	0.12	0.0057**
Shade*Tree isolation	−0.196	0.104	0.0587
(B) Full model: Hyenas ~ habitat, prey, lions, cheetahs			
Presence model: binomial with logit link			
(Intercept)	−0.91	1.13	0.421
Shade	−0.153	0.412	0.711
Tree isolation	0.128	0.536	0.812
Percent Cover	−0.055	0.365	0.881
Distance to river	−0.55	0.399	0.168
Distance to confluence	0.255	0.434	0.556
Distance to kopje	0.057	0.329	0.864
Habitat	0.058	0.411	0.888
Buffalo	−0.201	0.987	0.838
Wildebeest	1.485	1.265	0.24
T. gazelle	2.066	0.822	0.012*
Lion	1.141	0.696	0.101
Cheetah	0.932	0.869	0.284
Shade*Tree isolation	0.238	0.431	0.582
Count model: truncated Poisson with log link			
(Intercept)	−3.626	0.025	<0.0001***
Shade	−0.138	0.027	<0.0001***
Tree isolation	0.02	0.034	0.56591
Percent Cover	0.207	0.022	<0.0001***
Distance to river	0.065	0.033	0.0529
Distance to confluence	−0.012	0.034	0.7358
Distance to kopje	−0.071	0.023	0.0025**
Habitat	−0.02	0.028	0.47593
Buffalo/day	0.069	0.022	0.00163**
Wildebeest/day	0.136	0.021	<.0001***
T. gazelle/day	0.331	0.022	<0.0001***
Lions/day	0.485	0.04	<0.0001***
Lions/day^2^	−0.059	0.008	<0.0001***
Cheetahs/day	−0.01	0.057	0.86247
Cheetahs/day^2^	0.011	0.015	0.4671
Shade*Tree isolation	−0.013	0.028	0.63756
(C) Full model: Lions ~ habitat, prey, hyenas, cheetahs			
Presence model: binomial with logit link			
(Intercept)	−2.001	1.014	0.04839*
Shade	0.159	0.231	0.4916
Tree isolation	0.253	0.275	0.35792
Percent Cover	−0.261	0.199	0.18974
Distance to river	−0.459	0.249	0.06523
Distance to confluence	0.624	0.263	0.01791*
Distance to kopje	−0.26	0.173	0.13265
Habitat	0.2	0.228	0.38122
Buffalo	1.427	0.508	0.00496**
Wildebeest	0.13	0.989	0.89519
T. gazelle	0.629	0.613	0.30458
Hyena	1.229	0.699	0.07849.
Cheetah	0.579	0.41	0.15745
Shade*Tree isolation	0.242	0.231	0.29481
Count model: truncated Poisson with log link			
(Intercept)	−5.039	0.059	<0.0001***
Shade	−0.089	0.061	0.14963
Tree isolation	0.372	0.066	<0.0001***
Percent Cover	−0.15	0.057	0.00911**
Distance to river	0.015	0.062	0.80262
Distance to confluence	−0.199	0.069	0.00384**
Distance to kopje	0.068	0.053	0.19691
Habitat	−0.071	0.066	0.28409
Buffalo/day	0.344	0.028	<0.0001***
Wildebeest/day	0.114	0.053	0.03145*
T. gazelle/day	−0.433	0.098	<.0001***
Cheetahs/day	0.314	0.109	0.00380**
Cheetahs/day^2^	−0.004	0.029	0.88546
Hyenas/day	0.587	0.07	<0.0001***
Hyenas/day^2^	−0.032	0.012	0.00803***
Shade*Tree isolation	0.19	0.059	0.00122**

* indicates <0.05, ** indicates <0.01, and *** indicates <0.001

**Table 2:** Analysis of deviance. ANODEV is calculated as the difference in model deviance (−2logL) between the null model and the current model, divided by the difference between the null model and the full model. The incremental change in ANODEV contributed by submodels reflects the relative amount of residual variation explained by considering each predator species. *Incremental deviance* reported here reflects the difference between the current model and the habitat/prey model, thus reflecting the explanatory power of including (a) subordinate predator only, (b) top predator only, and (c) both subordinate and top predator

Models	ANODEV (%)	Incremental deviance
Cheetahs		
Null model	0.0	–
Habitat, prey	88.0	–
Habitat, prey, hyenas	91.4	3.4
Habitat, prey, lions	96.6	8.6
Habitat, prey, hyenas, lions	100.0	12.0
Hyenas		
Null model	0.0	–
Habitat, prey	78.2	–
Habitat, prey, cheetahs	81.1	2.9
Habitat, prey, lions	99.7	21.5
Habitat, prey, cheetahs, lions	100.0	21.8

Hyena presence and abundance were highest at sites with high localized prey availability (Table 1b), and abundance was higher at sites with increased tree cover and lower shade that were farther from kopjes. Hyena sightings increased with lion numbers up to a threshold of about 0.06 lion sightings per day, declining slightly at sites with highest lion numbers (Figure 3). Including lions as a predictor explained an incremental 21.5% of deviance in the hyena spatial models (Table 2).

### 3.2 Temporal activity patterns

**Figure 4:**
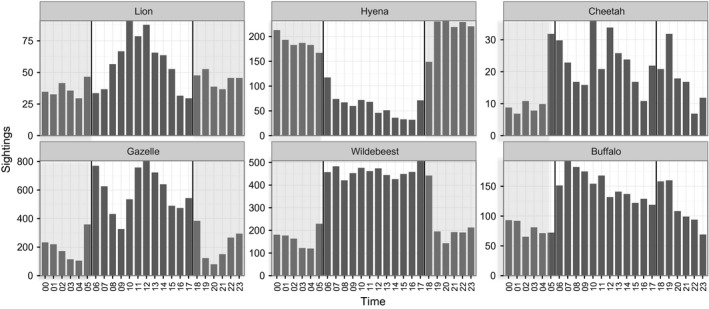
Temporal detection patterns. Temporal detection patterns calculated as the number of independent photographs per species per hour of the day. Shaded regions indicate nighttime hours. Daytime capture rates of lions and cheetahs reflect their use of shady trees and kopjes as daytime resting spots

All species except spotted hyenas were detected primarily during the day (Figure 4), and hourly activity patterns were positively correlated (*r *= 0.46–0.87) for all species pairs except those involving hyenas (*r *= −0.90 to −0.40). Lion and cheetah sightings occurred most frequently during midday, reflecting their use of shaded sites as daytime resting spots (Swanson, 2014); however, cheetahs were less active at night but exhibited activity spikes at dawn and dusk, whereas lions remained moderately active at all hours (Figure 4). In contrast, hyenas were detected primarily at night, and were most often observed moving, although they sometimes utilized trees or kopjes as daytime resting sites. All prey species were primarily diurnal, although buffalo were more likely to remain active into early nocturnal hours and gazelles demonstrated multiple activity peaks at dawn, midday, and dusk and a smaller peak near midnight (Figure 4). Thus, camera traps appear to reflect fine‐scale selection of shaded resting spots for lions and cheetahs during midday, but capture more general activity patterns throughout the remainder of the day.

### 3.3 Time since prey

**Figure 5:**
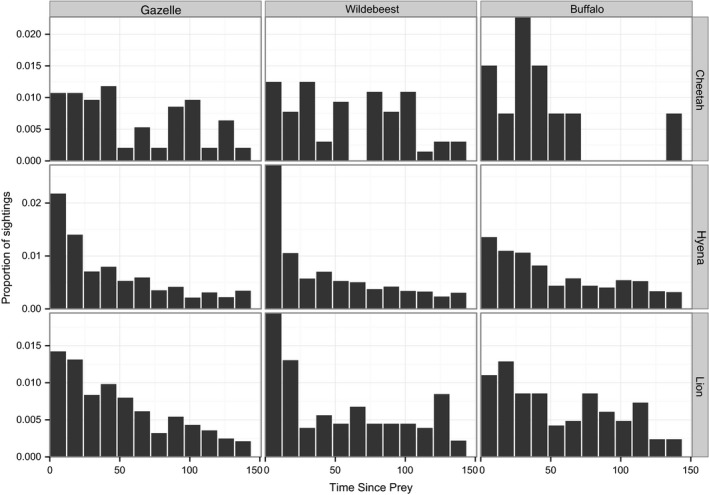
Short‐term temporal response to prey. Total number of cheetah, hyena, and lion sightings per 12‐hr period following prey sightings, aggregated across all camera‐trap sites. Histograms are faceted such that columns represent the first species seen and rows represent the species that follows. For example, the first chart in the first row shows number of cheetahs seen per 12‐hr period after a Thomson gazelle sighting

Lion and hyena activity was 1.5‐ to 3.5‐fold greater within the first 12–24 hr after prey sightings (Table 3; Figure 5); cheetahs exhibited a similar boost in activity during the first 12 hr after prey sightings, but relationships were insignificant, presumably due to smaller sample sizes. For all three predators, prey relationships were strongest for wildebeest during the first 12 hr, and then declined rapidly over the next 24 hr, suggesting that all three predators actively tracked wildebeest. Hyena exhibited a similar pattern with gazelle, whereas lion and cheetah activity declined more slowly following gazelle activity. Activity patterns for all three predators were higher for up to 36 hr following buffalo activity, but relationships were generally weaker than for other prey (Table 3).

**Table 3:** Short‐term avoidance and co‐occurrence. Observed versus expected (O/E) observations of predators in the first 0–12 and 12–24 hr since last detecting major prey species (Thomson's gazelle, wildebeest, buffalo) or other predators (lion, spotted hyena, cheetah). Ratios greater than 1 indicate positive associations (tracking), and ratios <1 indicate avoidance. Expected values were adjusted for diel activity patterns, and significant chi‐squared values (χ^2^ = (*O* − *E*)^2^/*E*) > 3.84 are indicated in bold

Species seen	Since	0–12 hr		12–24 hr	
		*O*/*E*	χ^2^	*O*/*E*	χ^2^
Lion	Gazelle	1.72	**11.63**	1.97	**17.10**
	Wildebeest	2.45	**29.25**	1.96	**10.90**
	Buffalo	1.52	3.21	1.78	**7.11**
	Hyena	2.20	**33.38**	1.53	**8.93**
	Cheetah	0.89	0.09	1.84	**4.22**
Hyena	Gazelle	2.44	**229.64**	1.97	**82.22**
	Wildebeest	3.23	**356.16**	1.54	**17.36**
	Buffalo	1.91	**34.10**	1.78	**11.31**
	Lion	2.29	**45.09**	1.39	3.66
	Cheetah	2.10	**9.20**	0.59	1.15
Cheetah	Gazelle	1.29	0.64	1.64	2.49
	Wildebeest	1.55	1.56	1.18	0.14
	Buffalo	2.15	1.23	1.06	0.00
	Lion	0.00	**5.34**	1.09	0.04
	Hyena	1.60	2.03	0.60	1.33

### 3.4 Time since predator

**Figure 6:**
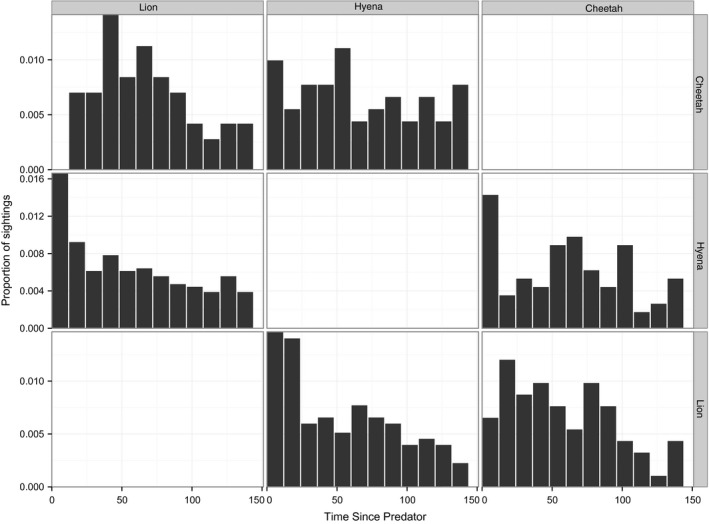
Short‐term temporal response to predators. Total number of cheetah, hyena, and lion sightings per 12‐hr period following predator sightings, aggregated across all camera‐trap sites. Histograms are faceted such that columns represent the first species seen and rows represent the species that follows. For example, the first chart in the first row shows number of cheetahs seen per 12‐hr period after a lion sighting

Cheetahs never appeared at sites within 12 hr of a lion sighting (Figure 6; Table 3), but showed expected levels of activity 12–36 hr after lion sightings and heightened activity 36–48 hr after a lion sighting (Figure 6; χ^2^ = 5.66, *p *= .017). By contrast, lions neither followed nor avoided cheetahs during the first 12 hr after a cheetah sighting (Table 3). Although cheetahs did not show any patterns of attraction or avoidance to hyenas, hyenas appeared at sites approximately 2.2 times more often than expected by chance in the first 12 hr after cheetah sightings (Table 3).

Lions and hyenas often appeared in the first hours after each other (Figure 6), and hyenas sometimes appeared while lions are still at the camera (three cases, all of which involved lions making and remaining on a kill). However, whereas lion sightings remained significantly higher for 24 hr following hyena sightings, hyena sightings declined sharply following 12 hr after a lion sighting (Table 3; Figure 6).

## 4 Discussion

Although subordinate competitors are expected to seek out “competition refuges” by selecting marginal habitats (Durant, 1998; Linnell & Strand, 2000), we found that interference competition among lions, hyenas, and cheetahs did not translate into long‐term displacement by subordinate species, even at fine spatial scales. In fact, hyenas and cheetahs were positively associated with lions except perhaps in areas with exceptionally high lion use; similarly, cheetahs showed no evidence of avoiding hyenas except in areas of extremely high hyena use. Fine‐scale temporal analyses further indicated that hyenas and lions actively tracked each other, whereas cheetahs actively avoided lion‐occupied areas for at least 12 hr. These contrasting patterns suggest that while cheetahs perceive lions as a threat, they are able to avoid them behaviorally, thus minimizing the need for long‐term spatial avoidance and the subsequent loss of access to resources.

### 4.1 Cheetahs

Our results challenge the long‐standing perception of cheetahs as “refuge species” that are only able to persist in marginal areas with low lion, hyena, and prey densities (e.g., Caro & Stoner, 2003; Chauvenet et al., 2011; Durant, 1998, 2000; Laurenson, 1994, 1995; Saleni, 2007). Lions and cheetahs were both attracted to shady trees on the open plains (Table 1a, c), and cheetahs continued to use these preferred habitat patches despite moderately high levels of lion use. Instead of generally avoiding areas of known lion use, cheetahs reduced the chance of encountering a lion by avoiding those areas only when lions were present (Figure 6). Such active temporal avoidance did not appear to reduce access by cheetahs to their primary prey (Figure 5), Thomson's gazelle. In contrast to their temporal avoidance of lions, cheetahs show no temporal avoidance of hyenas (Tables 1a and 2; Figure 6) perhaps because hyenas are less of a threat than lions (Laurenson, 1994, 1995) or because cursorial predators are expected to trigger smaller avoidance responses than ambush predators (Preisser, Orrock, & Schmitz, 2007; Schmitz et al., 2004).

By responding reactively, and only avoiding preferred habitats immediately following lion presence, cheetahs may minimize their risk of encountering lions while still maintaining access to vital resources. Indeed, Rostro‐García, Kamler, and Hunter (2015) found that cheetahs successfully utilized and hunted in a wider array of habitats than previously thought, and recent work by Broekhuis et al. (2013) and Vanak et al. (2013) found that cheetahs distributed themselves primarily with respect to their prey and only secondarily maintained a safe distance from the nearest lion. Our results confirm that this behavior does not translate into spatial displacement but is instead achieved by fleeting temporal avoidance at a given location (i.e., within 12 hr of a lion sighting). Such fine‐scale active avoidance may be key to cheetah persistence in the face of interference competition by lions: Lion density has no significant impact on cheetah numbers through time or across reserves (Mills & Mills, 2014; Swanson et al., 2014).

### 4.2 Hyenas

Despite dissimilar habitat preferences, lions and hyenas appear at the same camera‐trap sites (Figure 3) on the same days (Figure 6). These patterns of attraction could reflect active attraction between predator species: Lions and hyenas actively scavenge from each other (Kissui & Packer, 2004; Périquet et al., 2015), although interference outcomes are dependent on group size and population densities (Cooper, 1991; Höner et al., 2002; Watts & Holekamp, 2008). Although the quadratic relationship between lions and hyenas across camera sites (Figure 3) was significant (Table 1) and may reflect an attraction of hyenas to lions until some threshold lion density is reached, the effect size at high lion densities is small and driven exclusively by observations at three sites (out of 210 sites occupied hyenas).

Alternatively, the apparent attraction between lions and hyenas may be driven by mutual attraction to shared prey. Lions and hyenas both prey upon wildebeest (Hayward, 2006; Kruuk, 1972; Schaller, 1972), appear more often at sites with higher localized prey densities (Table 1b, c), and appear to actively follow these prey (Figure 5). Lion and hyena peak more sharply and decline more quickly following wildebeest sightings than sightings of each other (Table 3), but further investigation and additional data are needed to determine whether lions and hyenas are actively attracted to each other or simply tracking the same prey.

## 5 Conclusions

Aggressive interactions among predators are widespread and substantial (Arim & Marquet, 2004), triggering active avoidance by subordinate species to minimize these encounters (Prugh et al., 2009; Ritchie & Johnson, 2009). This avoidance is often assumed to result in the costly displacement from preferred habitats or reduced access to prey and result in population suppression (e.g., Durant, 1998, 2000). However, while these costs are documented in a range of systems (Schmitz et al., 1997; Swanson et al., 2014), our results suggest that active avoidance does not *universally* translate into costly large‐scale displacement associated with population suppression.

Specifically, we found that despite actively avoiding lions (Figure 6; see also Durant, 2000), cheetahs are displaced from neither preferred habitat patches nor larger areas across the landscape (Broekhuis et al., 2013; Swanson et al., 2014; Vanak et al., 2013), nor do they lose access to their prey (Figure 5) or show any signs of population‐level suppression by lions (Swanson et al., 2014). In sharp contrast, African wild dogs actively avoid lions (Webster, McNutt, & McComb, 2012), and this avoidance translates into large‐scale displacement that carries heavy demographic costs: Lions appear to directly suppress wild dog populations largely through exclusion from large areas of the landscape (Creel & Creel, 1996; Swanson et al., 2014).

Thus, the relative importance of this “landscape of fear” varies across systems and species and is likely dependent on a number of factors including hunting strategy and habitat complexity. For example, ambush predators trigger more severe avoidance responses than those with more cursorial approaches (Preisser et al., 2007; Schmitz et al., 2004), and avoidance costs are low for non‐habitat‐specialists (Preisser et al., 2007; Schmitz et al., 2004). Habitat complexity (Janssen, Sabelis, Magalhães, Montserrat, & Van der Hammen, 2007) and high ecosystem productivity (Elmhagen & Rushton, 2007; Elmhagen et al., 2010) are further expected to minimize displacement and suppression. Ultimately, the landscape of fear created by top predators is complex and species‐specific and does not always translate into population‐level suppression. The different patterns of spatial and temporal avoidance by subordinate species may help explain the diverse patterns of suppression and coexistence within predator guilds.

## Conflict of Interest

The authors declare no conflict of interests for this work.

## Data Accessibility

All data used in this study are available at datadryad.org/resource/doi:10.5061/dryad.5pt92.
