# Supplementary material for: In the absence of a “landscape of fear”: How lions, hyenas, and cheetahs coexist
Source: Ecol Evol. 2016 Nov 6;6(23):8534–45. doi: 10.1002/ece3.2569 (PMC5167031; doi:10.1002/ece3.2569)
Supplement: Supplementary file 1 [file ECE3-6-8534-s001.docx]

Table S1: Summaries of predictor variables prior to standardization.

|  | **mean** | **sd** | **median** | **minimum** | **maximum** | **n** |
| --- | --- | --- | --- | --- | --- | --- |
| Daily_lion | 0.007 | 0.013 | 0.003 | 0.000 | 0.094 | 225 |
| Daily_hyena | 0.032 | 0.052 | 0.018 | 0.000 | 0.613 | 225 |
| Daily_cheetah | 0.004 | 0.009 | 0.000 | 0.000 | 0.064 | 225 |
| Buffalo | 0.020 | 0.029 | 0.009 | 0.000 | 0.228 | 225 |
| Gazelle | 0.059 | 0.081 | 0.030 | 0.000 | 0.478 | 225 |
| Wildebeest | 0.037 | 0.030 | 0.032 | 0.000 | 0.221 | 225 |
| Shade | 2.098 | 1.306 | 2.000 | 0.000 | 4.000 | 225 |
| Grass | 2.107 | 1.068 | 2.000 | 0.000 | 4.000 | 225 |
| Tree isolation | 641.798 | 639.839 | 277.500 | 8.800 | 1500.000 | 225 |
| Distance to river | 2450.091 | 1869.475 | 2085.650 | 2.292 | 9147.744 | 225 |
| Distance to confluence | 2240.434 | 1443.258 | 1902.240 | 45.047 | 7838.165 | 225 |
| Distance to kopje | 3743.287 | 2306.004 | 3373.865 | 2.359 | 10218.652 | 225 |
| Habitat | 1.627 | 0.918 | 1.000 | 1.000 | 3.000 | 225 |
| Perecent Cover | 3.501 | 0.622 | 3.348 | 2.398 | 6.165 | 225 |
